# Mouse Models of Human GWAS Hits for Obesity and Diabetes in the Post Genomic Era: Time for Reevaluation

**DOI:** 10.3389/fendo.2017.00011

**Published:** 2017-02-07

**Authors:** Samantha Laber, Roger D. Cox

**Affiliations:** ^1^Mammalian Genetics Unit, Medical Research Council Harwell Institute, Oxfordshire, UK; ^2^Department of Physiology, Anatomy and Genetics, University of Oxford, Oxford, UK

**Keywords:** GWAS, epigenetics, obesity, cross-species conservation, genetic association

In recent years, genome-wide association studies (GWAS) have identified hundreds of loci and thousands of single-nucleotide polymorphisms (SNPs) associated with type 2 diabetes mellitus (T2DM) and obesity traits [such as body mass index (BMI) and waist–hip ratio (WHR)] in the human population (1–4). The vast majority of these SNPs are in non-coding regions of the genome and distal to promoters, suggesting they act through gene regulation which makes their functional interpretation challenging (5). Collectively, comparing the epigenetic landscape between mouse and human has established new pathways involved in obesity and diabetes, and in fact, inter-species conservation has successfully been used as criteria in finding functional and disease-relevant elements (6–8). By contrast, genome-wide comparative analysis of the mouse and human epigenome across tissues has highlighted the presence of cis-regulatory divergence (9, 10). New mouse engineering approaches together with bioinformatics dissection of trait-associated regions, for example, epigenetic modifications and genome interactions hold great promise to fully understand the underlying mechanisms of human disease-associated non-coding variants in T2DM and obesity.

## The Context-Specific Nature of Human GWA Signals in Human

Over 80% of loci identified by GWAS are in intergenic and intronic regions and many of these genetic risk regions are enriched for histone modifications (5), suggesting they act as regulatory elements which appear to function in a highly cell-selective manner. Due to the tissue specificity as well as the developmental and epigenetic complexity of gene regulation, functional approaches require the study of the relevant tissue and cell type as well as genetic and bioinformatics approaches that reliably assess the regulatory role of non-coding variants (7, 11, 12). Ongoing progress in high-throughput sequencing and the development of new experimental tools are greatly advancing our capacity to study chromatin biology and genome function. In particular, ChIP-seq allows identification of transcription factor binding sites and chromatin states; chromosome conformation capture-based techniques (including 3C, 4C, 5C, CaptureC, and HiC) allow the study of chromatin interactions; and DNase hypersensitivity or ATAC-seq can identify accessible chromatin (13–16). Additionally, tools like HaploReg (17), Enlight (18), RegulomeDB (19), and The Islet Regulome Browser (11) are emerging that allow the integration of GWAS results with genetic and epigenetic annotations that can be used to dissect the gene regulatory networks that underpin genomic association signals.

By integrating the information gained from functional genomics efforts such as the ENCODE (5) and Roadmap Epigenomics projects (20) together with expression quantitative trait loci (eQTL) results and functional studies, it becomes increasingly clear that adipose tissue is one of several key effectors of genetic risk loci for T2DM and obesity trait associations, particularly for WHR signals (2, 7, 21, 22). However, there is currently still a lack of comprehensive maps linking distal elements that harbor disease-associated variants with their target genes in relevant tissues and developmental stages. Furthermore, extensive fine-mapping of risk associations is crucial in order to narrow down the association signal to the likely causative variants which can then be functionally investigated (23). This has resulted in many studies being performed assuming that the closest gene to a given disease-associated signal is the causative one. Traditionally, target genes based on proximity to a signal were selected to model in the mouse using global or tissue-specific gene knockout or overexpression alleles to characterize gene function (24). However, with this approach, many target genes for GWA signals have potentially been overlooked, for example, in the case of the BMI-associated variants in *Fto* (25, 26). An additional level of complexity comes with the possibility for an association signal—that usually harbors dozens of SNPs—to potentially contain a number of disease-causing variants that might act in different tissues and/or at different times, affecting different genes. For example, there is currently evidence for intronic *FTO* risk variants to alter the expression of nearby genes in both adipose tissue and brain. An eQTL in human cerebellum links re9930509 to altered *IRX3* expression (25), rs1421085 has very convincingly been shown to be located within an enhancer for *IRX3* and *IRX5* in adipocyte precursors (7, 26) and rs1421085 and rs8050136 have been proposed to selectively alter *FTO* and *RPGRIP1L* expression in human-induced pluripotent stem cell-derived neurons (27). Therefore, mouse models which could help pinpoint variants, target genes, and relevant tissues would prove invaluable in the mechanistic dissection of human disease-associated sequence variants. However, whether it is possible to use the mouse for modeling regulatory variants (which is essential to capture the relevant spatiotemporal effects) will depend on the functional conservation of the regulatory circuitry of a given signal in human and mouse.

## Conservation Between Human and Mouse

It is estimated that our last common ancestor with the mouse was about 90 million years ago (28). At this point, many of the core physiological regulatory mechanisms had evolved, for example, mouse and human share the same basic mechanisms for controlling food intake *via* leptin and hypothalamic anorexigenic and orexigeneic neurons, and similarly insulin and glucagon are core effectors in glucose homeostasis. However, there have clearly been many evolutionary changes over this long period of time. At the level of the genome, chromosome number and organization have changed, although it is striking how large tracts of DNA have conserved their order of genes and show high coding sequence conservation (29). Thus, if we wish to use the mouse as model of human metabolic disease we can rely on much of the core conservation of ancient metabolic pathways and their regulation but cannot ignore the fact of their continued evolution that adapts and changes these mechanisms for the survival of two very different organisms. The mouse ENCODE Consortium reported that comparative gene expression data from human and mouse reveals that some sets of genes tend to cluster more by species than by tissue and *vice versa* (29). More recently, it has been suggested that gene clustering by tissue rather than species is much stronger than originally thought (30). Interestingly, single-cell sequencing of human and mouse pancreatic alpha and beta cells showed good cross-species correlation of transcriptomes although with some important species differences (31). Finally, Breschi et al. (32) describe how transcriptomes show a continuum of variation from species dominated clustering to organ dominated clustering. Importantly, for modeling GWA signals in other species, genes that varied little between species (and are more organ-specific) are more likely to overlap with human risk variants (32).

## Epigenomic Conservation Between Human and Mouse—Insights from the Mouse Encode Consortium

Some of the other key findings of ENCODE in the mouse genome were that human and mouse trans-regulatory networks (transcription factor networks) are considerably more conserved than the cis-regulatory landscape, which in fact accounts for the majority of regulatory plasticity between human and mouse (28, 29). At the same time, the degree of divergence of regulatory elements varies widely between different types of elements that are active in different tissue contexts (9, 28). The Mouse ENCODE Consortium (29) demonstrated that 79.3% of mouse candidate enhancers (predicted by patterns of histone modifications) and 66.7% of transcription factor binding sites have sequence orthologs in humans. Further, 61.5% of tested candidate mouse-specific enhancers also show enhancer activity in human embryonic stem cells in a reporter assay (29), suggesting a degree of functional conservation between human and mouse gene regulation. Based on this level of conservation, it is intriguing to ask the question whether mouse chromatin states could be used to identify potential sites for functional characterization in mouse for human GWAS hits. Mapping 4,265 SNPs from human GWAS studies onto the mouse genome using 15 mouse samples revealed that human GWAS hits are associated with specific chromatin states in relevant mouse tissues (29). For example, in mouse kidney, H3K4me1 is enriched in specific GWAS hits associated with urate levels and metabolites. For mouse liver-specific H3K36me3, GWAS hits related to HDL cholesterol and triglyceride levels are enriched. Together, 55% of mapped SNPs overlapped with at least one histone mark in mouse (29). These results suggest that histone modification marks can be used to inform about human risk variants and for the identification of candidate functional sequences for characterization of human GWAS hits in mouse.

Furthermore, SNPs with high regulatory potential are enriched in conserved transcription factor binding sites (19). Cheng et al. (33) show that conserved sequences occupied by orthologous transcription factors in human and mouse are enriched for GWAS variants. When investigating whether this is true for individual phenotypes, they found that SNPs associated with type I diabetes and several other traits are significantly enriched in conserved transcription factor binding sites, with 13 out of 20 type 1 diabetes SNPs being in conserved binding sites. By contrast, all of the SNPs associated with pulmonary function were found to be human-specific, suggesting that besides GWAS SNPs generally being enriched in conserved regulatory elements, that this enrichment is dependent on the trait (33). Whether this is the case for T2DM and obesity traits association is yet to be investigated. With continuous efforts and the increase in available mouse genome data sets, it will become possible to draw conclusions about the human–mouse conservation of transcription factor occupancy and enrichment of GWAS SNPs in adipose tissue. Indeed, on a cellular level, a systematic comparison between the human and mouse epigenome during adipocyte development and in different fat depots is largely missing, and Mouse ENCODE has currently only limited adipose tissue datasets that could be matched to human. Though, Mikkelsen et al. (34) generated a comparative analysis of chromatin state maps together with gene expression profiles from human adipose tissue and mouse 3T3-L1 at four time points during differentiation. They showed that although a significant amount of open chromatin in orthologous regions were shared between the two models (15–30%), most of them were species-specific. While we are not proposing that this affected the key findings of this study, it is worth pointing out that comparing a mouse cell line and primary human tissue-derived pre-adipocytes with their accompanying ontogenetic differences can potentially hinder the interpretation when using these data sets for dissecting specific GWAS loci with the aim to establish relevant functional sites.

Taken together, although the cis-regulatory landscape has substantially diverged between human and mouse on a global level, human trait-associated SNPs are enriched in sites that are conserved between the two species for the majority of traits investigated.

## The Potential for New Strategies in Mouse Model Engineering

**Figure 1:**
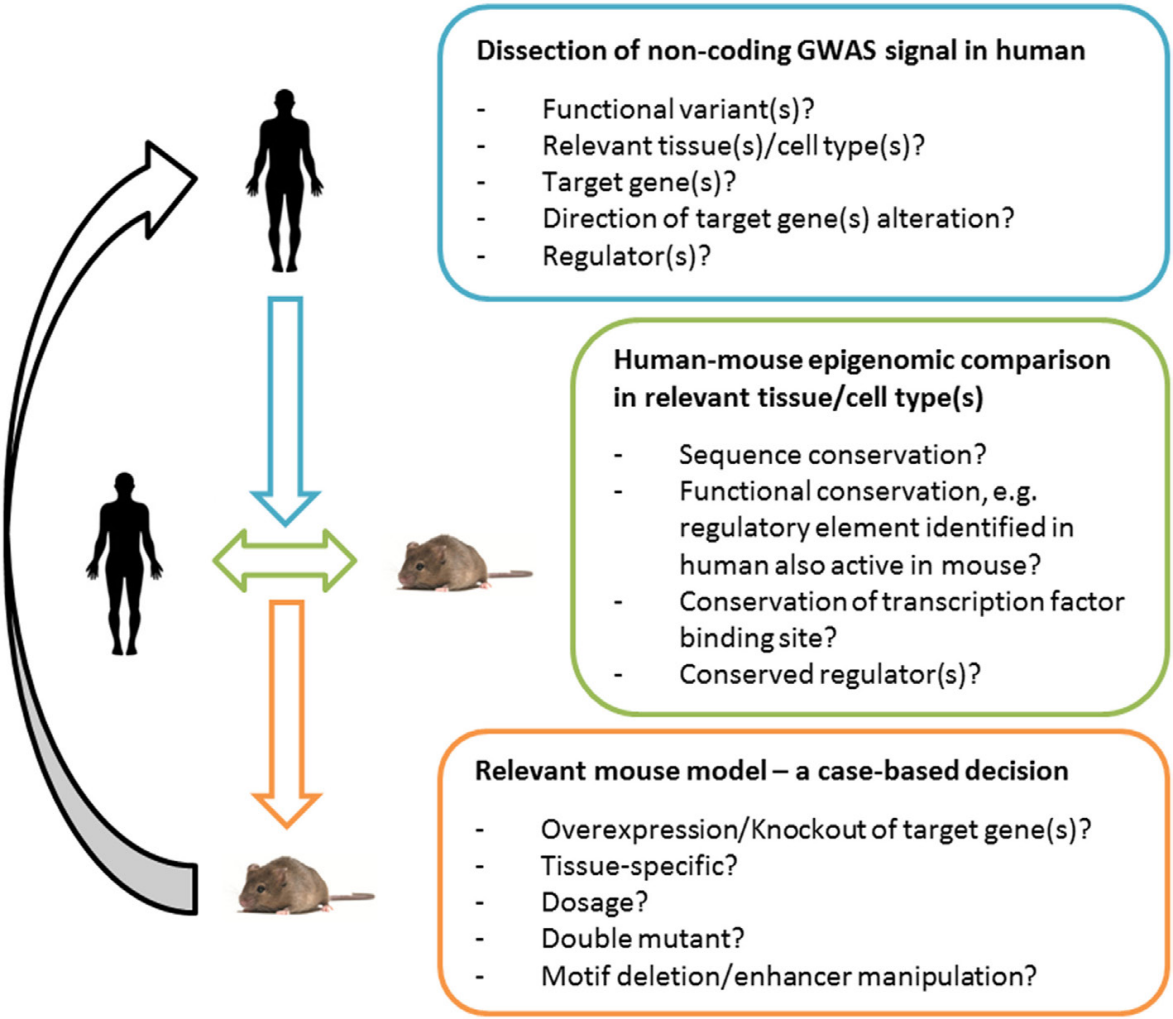
**Functional validation of variants in human and mouse**. An integrative approach for the generation of meaningful and informative mouse models of human Genome-Wide Association Study (GWAS) signals. Deciphering the underpinnings of an association signal in the human context is essential. For mechanistic studies *in vivo*, a human-to-mouse epigenomic comparison can guide the choice of a relevant mouse model, e.g., in the case of low or insufficient functional conservation of a regulatory site (or the lack of data sets that can determine the latter) a classical tissue-specific target gene manipulation can prove valuable; in the case of a high functional conservation (based on genomics and bioinformatics dissection of the loci), a model that selectively manipulates the regulatory region can in principle be useful. Comparing the human–mouse epigenome can be expected to become increasingly powerful with the improvement of quality as well as the comprehensiveness of genomic data sets and tools. The translational utility will depend on the mouse model chosen and the information gained can feedback and help interpret human GWAS signals.

With our current knowledge of the context-specificity of gene regulation and consequently the many layers of complexity of most GWAS signals, it becomes increasingly clear that it is necessary to study and understand the underlying regulatory network in the relevant human tissue (Figure 1). In the past, a successful approach to studying the function of individual candidate genes *in vivo* has been achieved by generating global knockout and overexpression models (24). However, these models do not resemble the tissue-specific nature of alterations in regulatory elements. Tissue-specific target gene manipulation using CRE drivers can be a powerful tool to overcome this problem. However, another challenge comes with the current lack of reliable pre-adipocyte-specific CRE lines that can be used to assess the tissue-specific effect of identified target genes in cases of pre-adipocyte-specific signals. Recent advances in genome engineering, namely CRISPR/Cas9, opened the opportunity to conveniently alter any regulatory sequence of interest (35). In other words, it is now possible to genome edit transcription factor binding sites and enhancer elements in the mouse which in principle has the potential to create mouse models of human risk variants that (i) are cell type-specific; (ii) alter all target genes; (iii) alter target genes at the relevant level and direction; and (iv) alter target genes at the relevant time of development (Figure 1).

## Conclusion and Future Directions

The majority of human genetic variants associated with common metabolic disease traits are located within distal regulatory elements. With our current knowledge of gene regulation and the context-specificity of the signal, it is necessary to understand the signal in human. Identifying targets and context is crucial in engineering a *relevant* mouse model. A comprehensive human-to-mouse epigenomic comparison can be informative about human risk variants. Although intriguingly, whether manipulation of regulatory elements will become a tool to dissect human obesity/T2DM risk variants in the mouse will depend on the functional conservation of a given signal. This is yet to be established and offers an exciting avenue to explore.

## Glossary

**ATAC-seq**—assay for transposase-accessible chromatin followed by high-throughput sequencing. This technique allows the identification of open chromatin.

**BMI**—body mass index. A measure of body weight that takes account of an individual’s size and calculated by dividing body weight by height squared.

**ChIP-seq**—chromatin immunoprecipitation followed by high-throughput sequencing. This technique allows the identification of DNA fragments that are bound by a specific antibody.

**Cis-regulatory**—non-coding DNA sequences in or near a gene required for its spatiotemporal expression that characteristically contain transcription factor binding sites.

**CRE**—Cre recombinase recognizes DNA sequences known as LoxP sites and when a pair of sites is provided in the same orientation this leads to deletion of the intervening sequence. In this way, a segment of DNA such as a key exon (said to be floxed) can be deleted resulting in, for example, a null mutation. This can be done *in vivo* by gene editing to place LoxP sites in the required location and then crossing animals that carry this modification to Cre recombinase strains, which then results in recombination. The expression of Cre recombinase can be driven by a promoter of choice either as a transgene or knocked into an endogenous gene promoter. Thus, the recombinase can be expressed in specific tissues as required allowing cell- or tissue-specific recombination, i.e., for the generation of a conditional knockout.

**Epigenome**—a network of chemical compounds (for example, DNA methylation or histone modifications) surrounding DNA that modify the genome without altering the DNA sequence itself. These modifying elements play a role in determining which genes are active in a particular cell at a particular time.

**eQTL**—expression quantitative trait loci are genomic loci that contribute to variation in the expression levels of mRNAs. For example, in individuals in a population inheriting SNP allele A, the expression of gene Y is found to be quantitatively increased or decreased on average relative to the other SNP alleles inherited across the population assayed. This is a correlated trait rather than a direct functional link between a SNP and the expression of a gene. Further, any particular SNP marks a haplotype (a linked co-inherited group) of SNPs and as such represents a locus.

**GWAS**—Genome-Wide Association Study.

**iPSC**—induced pluripotent stem cell.

**SNP**—single-nucleotide polymorphism.

**T2DM**—type 2 diabetes mellitus.

**Transcriptome**—the entire mRNA expressed from the genes of a cell.

**Trans-regulatory**—in the context of transcriptional regulation, a trans-acting element is usually a DNA sequence that contains a gene. This gene encodes for a protein (or other molecules such as microRNA) that will regulate another target gene.

**WHR**—waist-to-hip ratio.

## Author Contributions

SL and RC wrote, edited, and approved the manuscript.

## Conflict of Interest Statement

The authors declare that the research was conducted in the absence of any commercial or financial relationships that could be construed as a potential conflict of interest.
